# The prognostic value of serum amyloid A in solid tumors: a meta-analysis

**DOI:** 10.1186/s12935-019-0783-4

**Published:** 2019-03-20

**Authors:** Hai-yingjie Lin, Guo-qiang Tan, Yan Liu, Shao-qiang Lin

**Affiliations:** 1 Department of Orthopedics, The Third Affiliated Hospital of Southern Medical University, Guangzhou, 510630 Guangdong China; 2 Department of Oncology, Jiangmen Central Hospital, Jiangmen, 529030 Guangdong China; 3 Department of Oncology, Affiliated Dongfeng Hospital, Hubei University of Medicine, Shiyan, 442008 Hubei China; 4 Clinical Department of Guangdong Metabolic Disease Research Center of Integrated Chinese and Western Medicine, The First Affiliated Hospital of Guangdong Pharmaceutical University, Guangzhou, 510080 Guangdong China

**Keywords:** Serum amyloid A, Solid tumors, Prognosis, Meta-analysis

## Abstract

**Background:**

Previous studies have demonstrated that serum amyloid A (SAA) levels are correlated with the clinical outcomes of solid tumors. However, the available data have not been systematically evaluated. The objective of the present meta-analysis was to explore the prognostic value of SAA levels in solid tumors.

**Methods:**

Eligible studies were identified from the PubMed, EMBASE and Science Citation Index electronic databases. The clinical characteristics, disease/progression-free survival (DFS/PFS) and overall survival (OS) were extracted from the eligible studies. The pooled hazard ratios (HRs) and 95% confidence intervals (CIs) were calculated with Stata 12.0 software. We also performed subgroup, meta-regression and sensitivity analyses.

**Results:**

In total, 12 eligible studies including 2749 patients were enrolled in the present meta-analysis. The pooled HRs with 95% CIs showed that elevated levels of SAA were significantly associated with poor OS (HR = 3.01, 95% CI 1.96–4.63) and DFS/PFS (HR = 1.67, 95% CI 1.31–2.12) in patients with solid tumors. Although publication bias was seem found in the studies with regard to OS, a further trim and fill analysis revealed that the adjusted HR was 3.02 (95% CI 1.96–4.63), which was close to the original HR. Subgroup analysis confirmed an elevated level of SAA as a strong prognostic marker in patients with solid tumors, regardless of tumor type, detection method, cut-off value, sample size, area and variance analyses.

**Conclusion:**

Our meta-analysis indicated that elevated levels of SAA might be an unfavorable prognostic marker for OS in patients with solid tumors.

## Introduction

Despite the efforts of the scientific community, cancer is still a serious public health problem worldwide. Based on GLOBOCAN estimates, approximately 14.1 million new cancer cases and 8.2 million cancer-related deaths occurred in 2012 worldwide [1]. Early diagnosis and treatment monitoring can improve the prognosis of cancer patients. However, most serum biomarkers are lack of sensitivity and specificity for cancer patients in early or localized disease [2]. Therefore, there is urgent need to identify a novel biomarker that can effectively monitor progression and predict prognosis in cancer patients.

Previous studies have proposed the concept that chronic inflammation promotes cancer development and progression [3, 4]. It has been widely accepted that tumor microenvironment is largely influenced by various inflammatory cells, which are key mediators of tumor growth, progression, and angiogenesis and metastasis [5]. Serum amyloid A (SAA) is an acute-phase, hepatic protein secreted in the course of acute infections and tissue damage, the expression of which is induced by several cytokines, including IL-1, IL-6, and tumor necrosis factor-α [6]. Research has demonstrated that the level of SAA may rapidly increase by up to 1000-fold in response to acute inflammation, and it is an ideal marker for inflammation in the body [7]. Furthermore, an elevated protein level of SAA protein is observed in cancer patients at an early stage, this finding has been identified both by immunochemistry and by proteomics methods in different common cancers, such as lung, ovarian, renal, uterine, nasopharyngeal cancer and in melanoma [8]. These results revealed that SAA may be as a potentially useful biomarker for cancer.

Meta-analysis has shown that a high level of C-reactive protein (CRP), another acute-phase protein, is significantly associated with the poor prognosis of some cancers, including esophageal, colorectal and urological cancers, and it has already been reported to be a prognostic marker in relevant cancers [9–11]. Latest meta-analysis suggests that high SAA levels were closely associated with a risk of developing cancer risk, but not to confirm their relation in terms of prognosis [12]. However, to the best of our knowledge, no meta-analysis has explored the prognostic value of SAA in cancer patients. Therefore, we performed the current quantitative meta-analysis to identify the prognostic significance of SAA levels in human solid tumors. These results will provide important information for personalized therapy.

## Materials and methods

### Search strategy

A comprehensive search strategy was employed to search PubMed, EMBASE and Science Citation Index up to February 2019 without applying a start date limit. The terms neoplasms, serum amyloid A protein, prognosis and cohort studies were used as medical subject headings (MeSH) and key words at the same time.

### Inclusion and exclusion criteria

Eligible studies in this meta-analysis met the following inclusion criteria: (1) patients were pathologically diagnosed with any type of solid tumors. (2) SAA as isolated from serum samples. (3) The study was designed as a cohort study. (4) The hazard ratios (HRs) with 95% confidence intervals (CIs) for survival outcomes were reported or could be calculated from the available data in the study.

The exclusion criteria were as follows: (1) the study did not report the prognostic value of SAA in solid tumors. (2) The study had a small sample size, with fewer than 50 patients. (3) The study was a case report, letter, conference abstract, review or duplicate article. (4) The article was not written in English.

### Quality assessment

Two independent reviewers separately assessed the quality of the studies using the Newcastle–Ottawa Scale (NOS), which addresses three aspects, namely, patient selection, study comparability and study endpoints. The maximum possible NOS score is 9. Studies that earned scores ≥ 5 were considered high quality, otherwise, they was considered low quality and removed. Any disagreement in the quality assessment of studies was settled by discussion.

### Data extraction and statistical analysis

The following information was extracted: surname of the first author, publication year, country, tumor types, clinical stage, patient number, methods of SAA detection, cut-off value, duration of follow-up, outcome, and variance analysis. Furthermore, HRs and 95% CIs were obtained directly from the eligible studies or estimated using the method suggested by Tierney et al. [13]. The above information was collected by two independent researchers, and any disagreement was resolved by group discussion and consensus.

All statistical analyses in the present meta-analysis were conducted using Stata 12.0 software. Pooled HRs and 95% CIs were used to evaluate the prognostic value of SAA levels in solid tumors. When the pooled HR was greater than 1, we concluded that an elevated level of SAA was a negative prognostic factor for patients. The heterogeneity of the pooled results was measured using Cochran’s *Q* test and the *I*^2^ statistic. Significant heterogeneity was defined as *P * < 0.1 or *I*^2^ > 50%. The random effects model was chosen to investigate the pooled HR when significant heterogeneity existed. Otherwise, the fixed effects model was used. To assess whether the results were influenced by other factors, subgroup, meta-regression and sensitivity analyses were conducted. Publication bias was tested by the funnel plot and Begg’s and Egger’s tests. If publication bias was found, a trim and fill analysis was used to evaluate the number of missing studies and recalculate the pooled risk estimate with the addition of those missing studies [14].

## Results

### Study selection

**Fig. 1:**
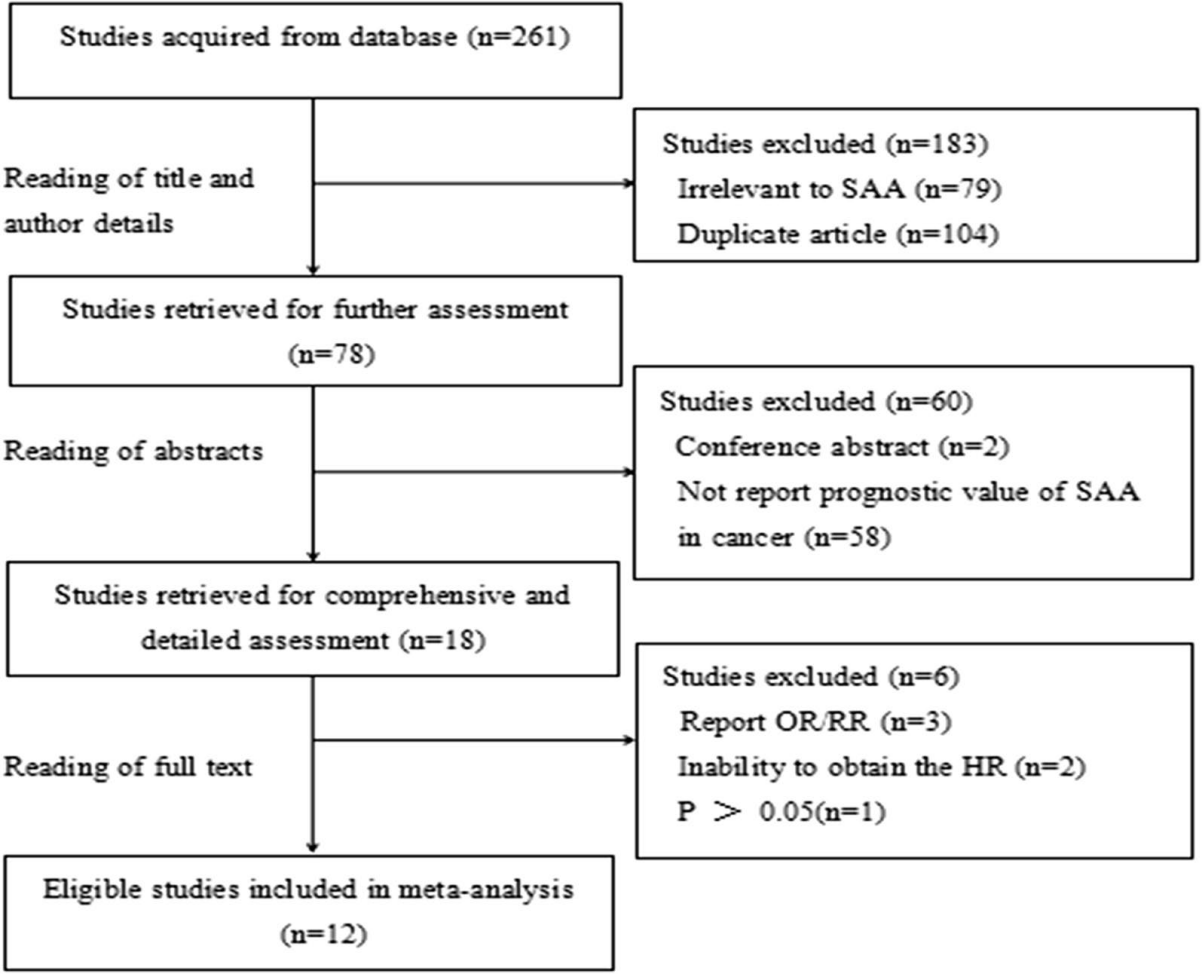
The flow chart of the study selection process in this meta-analysis

In total, 261 articles were initially collected by a systematic literature search of the PubMed, EMBASE and Science Citation Index electronic databases. By reading the titles and author details, 183 articles were excluded due to not involving SAA or being duplicate articles. The remaining 78 articles were further evaluated by inspecting the abstracts, and 60 articles were removed according to the exclusion criteria. In total, 18 studies were assessed by reading the full text, after which 6 studies were excluded because HRs could not be obtained from them. Finally, 12 eligible articles were included in this meta-analysis. The flow chart of the study selection process is shown in Fig. 1.

### Characteristics of the studies

In total, 12 eligible studies with 2749 patients published between 2000 and 2017 were included in the present meta-analysis. Seven studies were conducted in Asian countries (China, Japan and South Korea), and 5 were conducted in non-Asian countries (USA, UK, Holland and Germany). Nine different types of cancer were involved in the eligible studies, namely, renal cell carcinoma, breast cancer, gastric cancer, esophageal squamous cell carcinoma, rectal cancer, hepatocellular carcinoma, pancreatic cancer, nasopharyngeal carcinoma and non-small cell lung. The level of SAA in these studies was mostly measured using the nephelometry method, while enzyme-linked immunosorbent assays (ELISA) and latex agglutination turbidimetric immunoassays (LTIA) were also utilized. The cut-off value for the level of SAA was reported in 11 studies, and it ranged from 19.2 ng/ml to 22.0 mg/l. Among the included studies, OS was reported in 12 studies, and DFS/PFS was reported in 3 studies. The results of the studies were analysed by multivariate or univariate methods. Furthermore, only one study required extrapolation of the HR for OS, and the HRs and 95% CIs for both OS and DFS/PFS were directly reported in the other studies. The quality assessment scores of the included studies ranged from 5 to 9, all of which were regarded as high quality (Table 1). The detailed characteristics of the included studies are shown in Table 2.

**Table 1:** Newcastle–Ottawa Scale (NOS) scores for the quality assessment of the articles included in this meta-analysis

Study	Selection				Comparability		Outcome			Scores
	Representativeness of cases	Selection of controls	Ascertainment of cases	Outcome at start	Controls for the most important factor	Controls for additional factor	Assessment of outcome	Follow-up long enough for outcome	Integrity of follow-up	
Kimura [15]	1	1	1	1	1	1	–	1	–	7
Pierce [16]	1	1	1	1	1	1	1	1	1	9
Vermaat [17]	1	1	1	1	1	1	1	1	–	8
Wood [18]	1	1	–	1	1	1	–	1	–	6
Kwon [19]	1	1	1	1	1	1	–	1	–	7
Wang [20]	1	1	1	1	1	1	–	1	1	8
Meng [21]	1	1	1	1	1	1	1	1	1	9
Giessen [22]	1	1	1	1	1	1	1	1	–	8
Ni [23]	1	1	1	1	1	1	1	1	–	8
Haas [24]	1	1	1	1	–	–	1	1	–	6
Chen [25]	1	1	1	1	–	–	–	1	–	5
Zhao [26]	1	1	1	1	1	1	1	1	1	9

**Table 2:** Characteristics of the studies included in this meta-analysis

Study	Country	Tumor type	Clinical stage	N	Method	Cut-off value	Follow-up	Outcome	Variance analysis	HR
Kimura [15]	Japan	Renal cell carcinoma	I–IV	72	LTIA	8 mg/l	40.6 M (mean)	DSS^a^	Multivariate	Reported in text
Pierce [16]	USA	Breast cancer	0–III	734	Nephelometry	8.1 mg/l	24 M	OS	Multivariate	Reported in text
Vermaat [17]	Holland	Renal cell cancer	IV	114	ELISA	19.2 ng/ml	27.6 M (mean)	OS	Multivariate	Reported in text
Wood [18]	UK	Renal cell carcinoma	I–IV	119	Nephelometry	NR	2.52 Y (mean)	CSS^a^	Multivariate	Reported in text
Kwon [19]	South Korea	Gastric cancer	I–IV	115	LTIA	4.2 mg/l	9.3–88.8 M	OS	Multivariate	Reported in text
Wang [20]	China	Esophageal squamous cell carcinoma	I–IV	167	Nephelometry	8 mg/l	NR	OS	Multivariate	Reported in text
Meng [21]	China	Esophageal squamous cell carcinoma	I–IV	252	Nephelometry	8.18 mg/l	65.5 M (median)	OS	Multivariate	Reported in text
Giessen [22]	Germany	Rectal cancer	I–III	256	Nephelometry	5.3 mg/l	8.4 Y (median)	DFS/CSS^a^	Multivariate	Reported in text
Ni [23]	China	Hepatocellular carcinoma	NR	328	ELISA	7.5 µg/ml	3–32 M	DFS/OS	Multivariate/univariate	Extrapolated from data/reported in text
Haas [24]	Germany	Pancreatic cancer	NR	59	Nephelometry	22.0 mg/l	NR	OS	Univariate	Reported in text
Chen [25]	China	Nasopharyngeal carcinoma	I–IV	419	ELISA	4.28 mg/l	NR	PFS/OS	Univariate	Reported in text
Zhao [26]	China	Non-small cell lung	II–III	114	ELISA	101.4 µg/ml	24.3 M (median)	OS	Multivariate	Reported in text

*N* number, *M* month, *Y* year, *NR* not reported, *DSS* disease-specific survival, *CSS* cancer-specific survival, *OS* overall survival, *DFS* disease-free survival, *PFS* progression-free survival, *ELISA* enzyme-linked immunosorbent assays, *LTIA* latex agglutination turbidimetric immunoassay

^a^DSS and CSS both are regarded as OS

### Correlation between SAA level and survival outcome

**Fig. 2:**
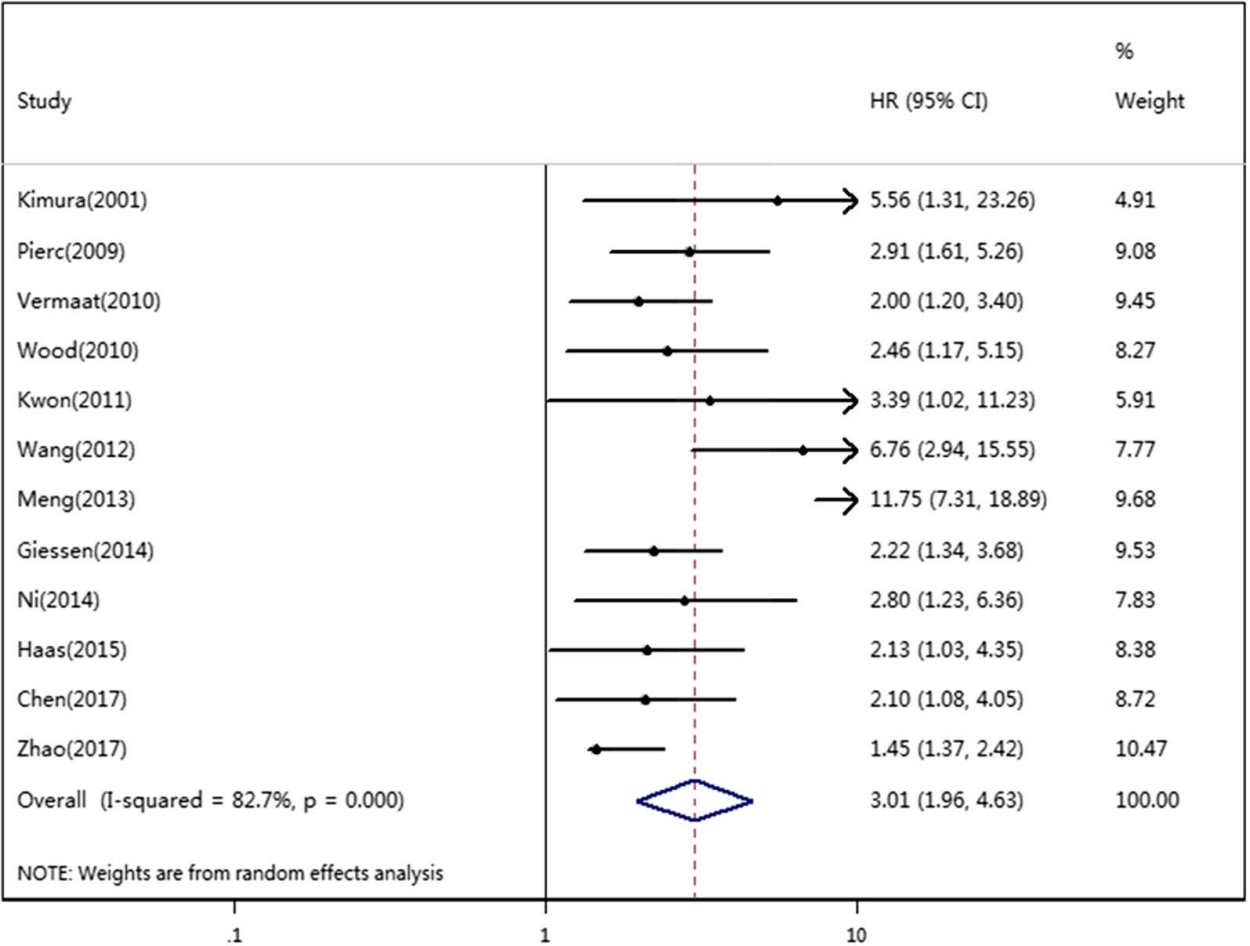
Forest plots of pooled HR of the relationship between SAA level and OS

**Fig. 3:**
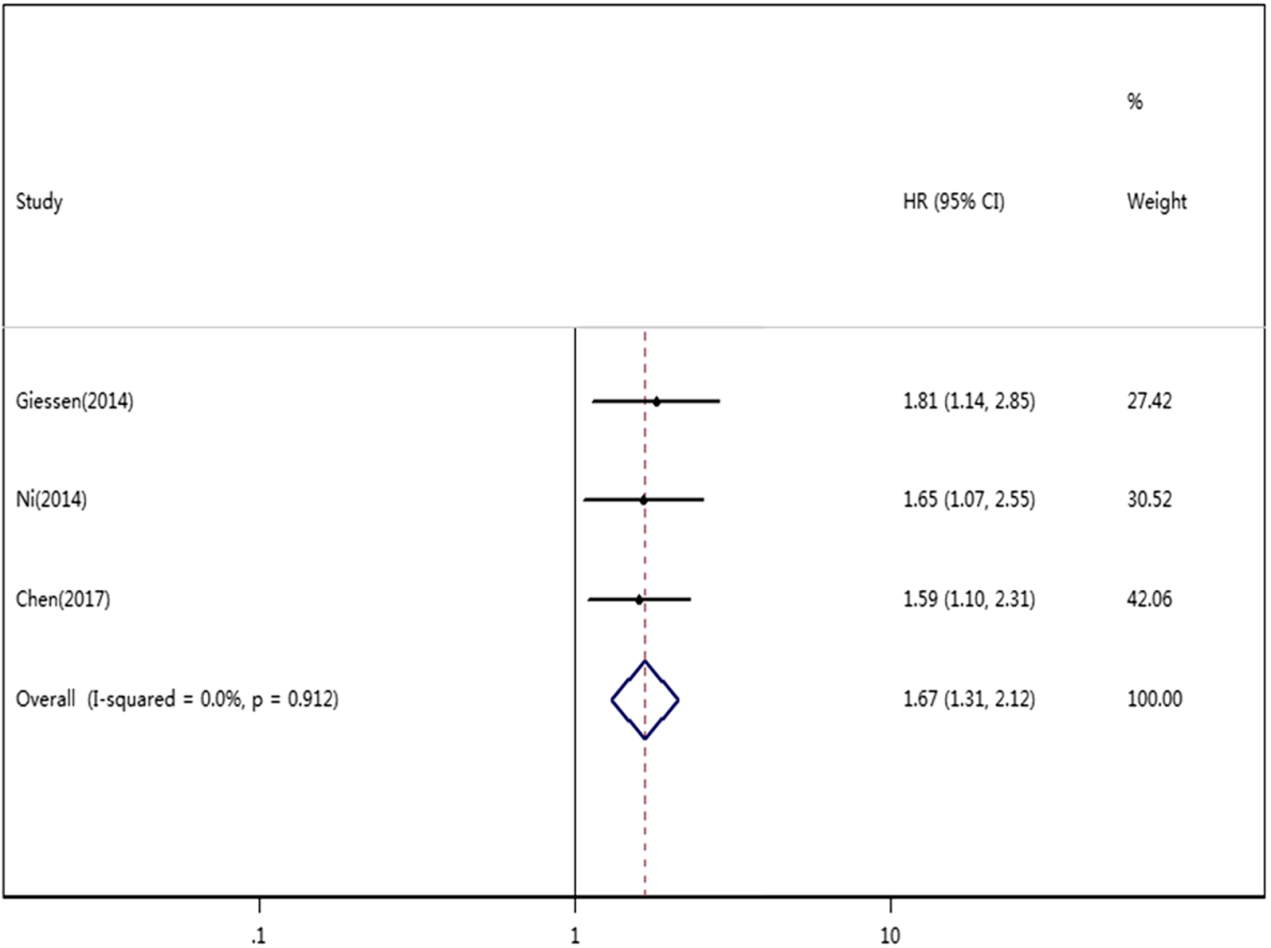
Forest plots of pooled HR of the relationship between SAA level and DFS/PFS

Twelve studies reported the relationship between SAA level and OS in a total of 2749 cancer patients. For OS, we calculated a pooled HR using a random effects model because significant heterogeneity was observed in this meta-analysis (*I*^2^ = 82.7%, *P *= 0.000). The pooled HR for OS was 3.01 (95% CI 1.96–4.63, *P *< 0.01), which suggested that an elevated level of SAA was significantly associated with poor OS in cancer patients (Fig. 2). Three studies reported the relationship between the SAA level and DFS/PFS in a total of 1003 cancer patients. For DFS/PFS, we calculated the pooled HR using a fixed effects model because no heterogeneity was observed in this meta-analysis (*I*^2^ = 0.0%, *P * = 0.912). The pooled HR for DFS/PFS was 1.67 (95% CI 1.31–2.12, *P *< 0.01), which also suggested that an elevated level of SAA was significantly associated with poor DFS/PFS in cancer patients (Fig. 3).

### Publication bias and sensitivity analysis

**Fig. 4:**
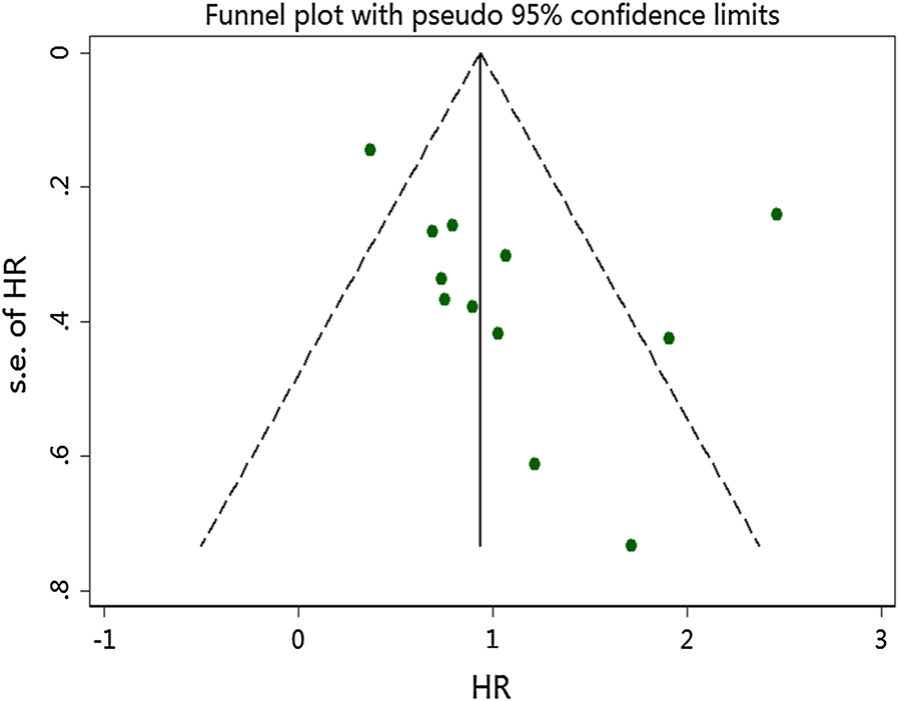
Funnel plots of publication bias of the relationship between SAA level and OS

**Fig. 5:**
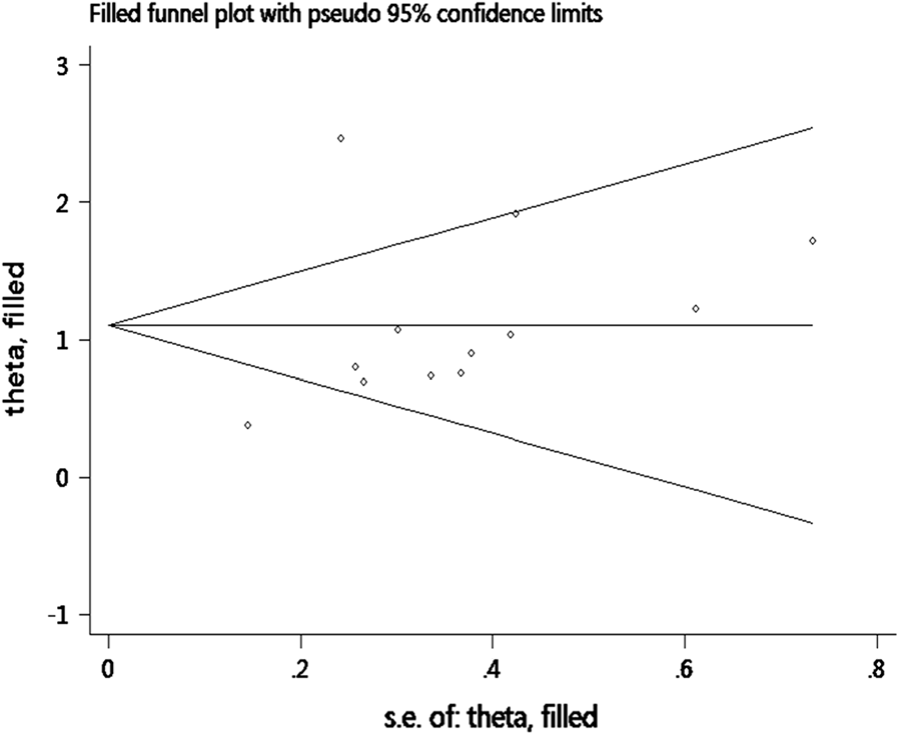
Funnel plots of trim and fill analysis of the relationship between SAA level and OS

**Fig. 6:**
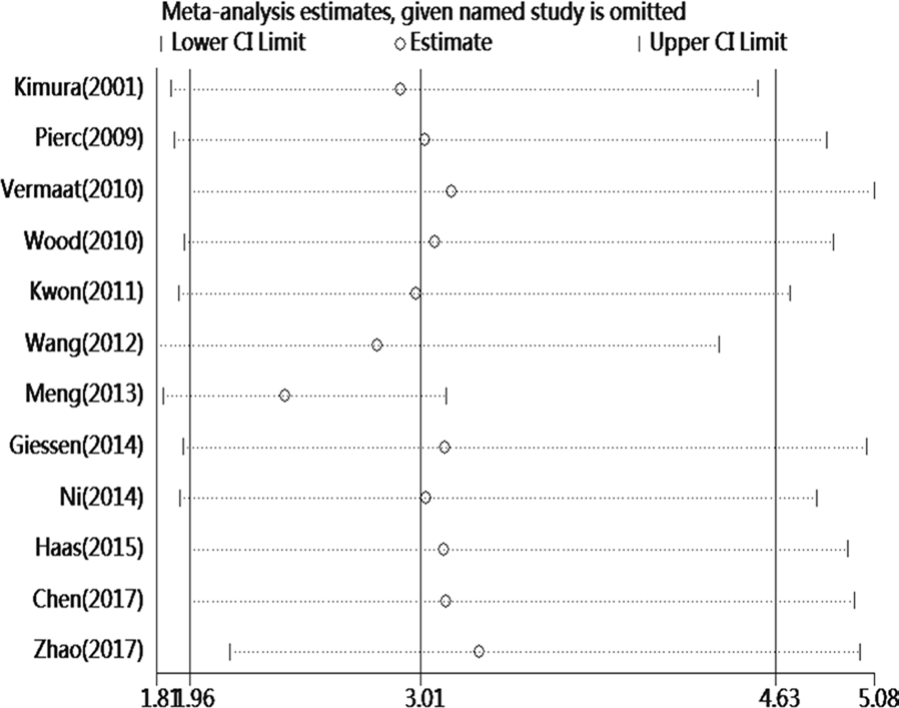
Sensitivity analysis of the relationship between SAA level and OS

For the OS, publication bias was revealed by asymmetrical funnel plots (Fig. 4). The result of Egger test’s was not significant (*P *= 0.214), but Begg’s test was significant (*P *= 0.047). Based on the trim and fill analysis for OS, no missing studies were imputed in the contour-enhanced funnel plots (Fig. 5). The analysis indicated that the imputed HR was 3.02 (95% CI 1.96–4.63), which had no influence on the overall effect of SAA level on OS. To assess whether the results were reliable, it was necessary to conduct further sensitivity analysis. After the removal of any single included study, the sensitivity analysis showed no significant change in the pooled estimates of the influence of the SAA level on the OS of patients with solid tumors (Fig. 6). For the DFS/PFS, the sample sizes were too small to conduct sensitivity and publication bias analyses.

### Subgroup and meta-regression analyses

For the OS, subgroup and meta-regression analyses were performed according to tumor types, detection methods, cut-off values, sample sizes, areas, and variance analyses. In the analysis stratified by tumor type, elevated levels of SAA were significantly correlated with poor OS in urinary cancers (HR = 2.31, 95% CI 1.54–3.48), digestive system cancers (HR = 3.97, 95% CI 1.98–7.94) and other cancers (HR = 1.70, 95% CI 1.34–2.16). When different detection methods were considered, elevated levels of SAA were significantly correlated with poor OS in the LTIA group (HR = 4.15, 95% CI 1.65–10.43), the nephelometry group (HR = 3.75, 95% CI 1.97–7.14) and the ELISA group (HR = 1.69, 95% CI 1.35–2.11). In the subgroup analysis by cut-off value, we found that poor OS was significantly correlated with a cut-off value less than or equal to the median value of 5.3 mg/l (HR = 1.80, 95% CI 1.47–2.20) and greater than 5.3 mg/l (HR = 4.87, 95% CI 2.32–10.26). A similar result was also observed in the subgroup analysis by sample size, both sample sizes less than or equal to a median value of 143 (HR = 1.77, 95% CI 1.42–2.20) and less than 143 (HR = 3.82, 95% CI 2.01–7.25) were significantly associated with OS. In addition, we also observed that elevated levels of SAA were associated with poor OS in studies conducted in Asian countries (HR = 3.78, 95% CI 1.72–8.32) and in non-Asian countries (HR = 2.30, 95% CI 1.76–3.00). Moreover, an elevated level of SAA was significantly associated with poor OS in multivariate analysis (HR = 3.32, 95% CI 1.91–5.77) and univariate analysis (HR = 2.27, 95% CI 1.50–3.45). More importantly, meta-regression analysis found that cut-off value had statistically significant in the inter-study heterogeneity (*P *= 0.03), indicating that cut-off value might help explain the sources of the inter-study heterogeneity. All results of the subgroup and meta-regression analyses for OS are shown in Table 3. However, For the DFS/PFS, the sample sizes were too small to conduct subgroup and meta-regression analyses.

**Table 3:** Subgroup and meta-regression analyses for OS in this meta-analysis

Subgroup	No. of studies	No. of patients	Fixed-effects model		Meta-regression	Heterogeneity	
			HR (95% CI)	*P*-value	*P* value	*I*^2^ (%)	*P*-value
All	12	2749	3.01 (1.96–4.63)	< 0.01		82.7	0.00
Tumor types					0.30		
Urinary system cancer	3	305	2.31 (1.54–3.48)	< 0.01		0.0	0.42
Digestive system cancer	6	1177	3.97 (1.98–7.94)	< 0.01		82.3	0.00
Other system cancer	3	1267	1.70 (1.34–2.16)	< 0.01		57.9	0.09
Detection methods					0.24		
LTIA	2	187	4.15 (1.65–10.43)	< 0.01		0.0	0.61
Nephelometry	6	1587	3.75 (1.97–7.14)	< 0.01		84.5	0.00
ELISA	4	975	1.69 (1.35–2.11)	< 0.01		11.2	0.34
Cut-off values (mg/l)					0.03		
≤ 5.3	6	1346	1.80 (1.47–2.20)	< 0.01		7.9	0.37
> 5.3	5	1284	4.87 (2.32–10.26)	< 0.01		81.1	0.00
Sample sizes					0.16		
≤ 143	6	593	1.77 (1.42–2.20)	< 0.01		25.0	0.25
> 143	6	2156	3.82 (2.01–7.25)	< 0.01		84.4	0.00
Areas					0.26		
Asian countries	7	1467	3.78 (1.72–8.32)	< 0.01		90.3	0.00
Non-Asian countries	5	1282	2.30 (1.76–3.00)	< 0.01		0.0	0.91
Variance analyses					0.46		
Multivariate	9	1943	3.32 (1.91–5.77)	< 0.01		87.3	0.00
Univariate	3	806	2.27 (1.50–3.45)	< 0.01		0.0	0.84

## Discussion

SAA is an acute-phase protein mainly produced by the liver under the regulation of inflammation-associated cytokines in the course of acute and chronic inflammatory processes. However, SAA is also synthesized in extrahepatic tissues, including primary and metastatic cancer cell lines [27, 28]. Previous studies have shown that SAA is an ideal biomarker for monitoring inflammation in many types of cancer [29]. Moreover, the sensitivity of SAA for the detection of the inflammatory response is considered to be tenfold higher than that of CRP [30]. Accumulated evidence demonstrates that tumor development is closely associated with chronic infection and inflammation. In 1979, there was already evidence that an elevated level of SSA was found in cancer patients [31]. Subsequently, SAA was proposed as a possible serum biomarker for many cancers, including renal cell cancer, breast cancer, gastric cancer, oesophageal cancer, rectal cancer, hepatocellular cancer, pancreatic cancer, and nasopharyngeal carcinoma [15–20, 22–25]. Clearly, SAA serves as a possible link between chronic inflammation and tumourigenesis, and elevated levels of SAA could contribute to tumor development and accelerate tumor progression and metastasis. However, because cancer is often considered a consequence of chronic inflammation, the consensus among many researchers is that SAA might influence tumor invasion through the extracellular matrix (ECM) by stimulating the production of matrix metalloproteinases (MMPs) [32]. Furthermore, SAA can modulate platelet adhesion and influence the adhesion of tumor cells to platelets, which may contribute to tumor invasion [32]. The precise mechanisms underlying the association of a high level of SAA with the development and progression of cancer are still poorly understood. Moreover, the prognostic role of SAA in solid tumors remains uncertain and needs to be addressed.

To the best of our knowledge, our study is the first meta-analysis assessing the prognostic role of SAA in various solid tumors. The meta-analysis included a total of 2749 patients with solid tumors, including renal cell carcinoma, breast cancer, gastric cancer, esophageal squamous cell carcinoma, rectal cancer, hepatocellular carcinoma, pancreatic cancer, nasopharyngeal carcinoma and non-small cell lung. In this meta-analysis, by estimating the pooled HR of the included studies, an elevated level of SAA was found to be significantly related to poor OS and DFS/PFS in patients with solid tumors. Although publication bias for OS was found in the meta-analysis, the results after adjustment by the trim and fill method were consistent with the original results. For the OS, further sensitivity analysis demonstrated that the result was not affected after excluding any single study. Finally, subgroup analysis between SAA levels and OS were performed, and an elevated level of SAA was still a negative marker for OS when the patients were stratified by tumor types, detection methods, cut-off values, sample sizes, areas, and variance analyses. Although inter-study heterogeneity was not significantly decreased in the subgroup analysis stratified by several factors, meta-regression analysis subsequently found that cut-off value maybe an important source of heterogeneity. Based on the above evidence, the results of our study are stable and reliable.

When stratified by tumor type, there was a trend for the association of increased SAA levels with poor OS to be the most sensitive for solid tumors in the digestive system, as the pooled HR of the subgroup with digestive system cancer was the highest of the entire group. However, this finding was not reported by previous studies. A similar result was observed in the subgroup analysis by cut-off value, the relationship between increased SAA level and poor OS was more sensitive for the subgroup with > 5.3 mg/l SAA than the subgroup with ≤ 5.3 mg/l SAA. This indicates that a greater tumor burden may aggravate the inflammatory response. Compared with ELISA, nephelometry showed greater sensitivity in predicting OS in the subgroup analyses by detection methods. Certainly, there is a need to confirm our results with further clinical trials.

However, some limitations in the current meta-analysis need to be acknowledged. First, only English language articles were included, while articles written in other languages were excluded. Second, one article could not directly provide a HR and its 95% CI. Thus, we extracted the HR and 95% CI through the procedure recommended by Tierney et al. [13], which may result in small statistical errors. Third, some studies provided HRs and 95% CIs from univariable analyses, which could lead to bias towards overestimation of the prognostic role of SAA, as the HRs in multivariable analyses may not be statistically significant after the consideration of other elements. Furthermore, the sample size of the included articles that reported DFS/PFS were too small to conduct publication bias, sensitivity, subgroup and meta-regression analyses, which might influence the stability of the corresponding results. Therefore, much more evidences need to confirm SAA can effectively predict DFS/PFS of patients with solid tumors. Finally, twelve studies in our meta-analysis both reported elevated levels of SAA are significantly associated with outcome in patients solid tumors. Latest article suggested that SAA maybe considered as a potential molecule to monitor the progression of acute lymphoblastic leukaemia [33]. However, until now, there are rare studies reported the relation between SAA and prognosis of patients with hematological malignancies. Thus we further recommend more clinical trials to determine prognostic value of SAA in hematological malignancies.

## Conclusion

In conclusion, the current meta-analysis showed that elevated levels of SAA are significantly associated with poor OS and DFS/PFS in patients with solid tumos. Furthermore, SAA might be used as a novel biomarker to predict OS of patients with solid tumors. In the future, larger-scale, multicentre and prospective studies are needed to validate our conclusions.
